# Discovery of chemically induced mutations in rice by TILLING

**DOI:** 10.1186/1471-2229-7-19

**Published:** 2007-04-11

**Authors:** Bradley J Till, Jennifer Cooper, Thomas H Tai, Peter Colowit, Elizabeth A Greene, Steven Henikoff, Luca Comai

**Affiliations:** 1 Fred Hutchinson Cancer Research Center, Seattle, WA 98107, USA; 2 Department of Biology, University of Washington, Box 355325, Seattle, WA 98195, USA; 3 USDA-ARS, Crops Pathology and Genetics Research Unit, Department of Plant Sciences, Davis, CA 95616, USA; 4 Section of Plant Biology and Genome Center, UC Davis, Davis, CA 95616, USA

## Abstract

**Background:**

Rice is both a food source for a majority of the world's population and an important model system. Available functional genomics resources include targeted insertion mutagenesis and transgenic tools. While these can be powerful, a non-transgenic, unbiased targeted mutagenesis method that can generate a range of allele types would add considerably to the analysis of the rice genome. TILLING (Targeting Induced Local Lesions in Genomes), a general reverse genetic technique that combines traditional mutagenesis with high throughput methods for mutation discovery, is such a method.

**Results:**

To apply TILLING to rice, we developed two mutagenized rice populations. One population was developed by treatment with the chemical mutagen ethyl methanesulphonate (EMS), and the other with a combination of sodium azide plus methyl-nitrosourea (Az-MNU). To find induced mutations, target regions of 0.7–1.5 kilobases were PCR amplified using gene specific primers labeled with fluorescent dyes. Heteroduplexes were formed through denaturation and annealing of PCR products, mismatches digested with a crude preparation of CEL I nuclease and cleaved fragments visualized using denaturing polyacrylamide gel electrophoresis. In 10 target genes screened, we identified 27 nucleotide changes in the EMS-treated population and 30 in the Az-MNU population.

**Conclusion:**

We estimate that the density of induced mutations is two- to threefold higher than previously reported rice populations (about 1/300 kb). By comparison to other plants used in public TILLING services, we conclude that the populations described here would be suitable for use in a large scale TILLING project.

## Background

Rice is both a food crop and a model system for scientific research. While the wild plant Arabidopsis has become the paramount model plant system, the spectrum of its traits cannot address fundamental questions of crop plant domestication and performance, therefore, an efficient experimental system based on a crop plant is needed. At the time of this publication, rice is the only crop for which complete genome sequence has been made available [1-3]. To realize the full potential of rice genomics, however, an appropriate genomic toolbox must be assembled. While methods such as expression profiling, proteomic and metabolomic analysis can provide insight into the function of genes and pathways, a complete analysis of function must involve disruption or modification of gene action.

Several approaches are available for the functional inactivation of rice genes. Tagging has been achieved with endogenous [4] and introduced transposons [5,6], and with the T-DNA of *Agrobacterium tumefaciens *[5,7]. The growing tagged gene databases will provide knock-outs for a majority of rice genes [5], but a substantial number will be missed. Such an outcome is exemplified by the tagging resources available in Arabidopsis [8], where even 360,000 tags leave more than 10% of the genes untagged [9]. While the probability of tagging a gene increases with the number of available tags, it does so asymptotically and with some sequence bias [10], and the efforts to extend the database produce diminishing returns.

Another method exploits RNA interference [11,12], triggered by expression of transgenic hairpin repeats homologous to the gene target. The efficacy of this approach, however, can vary [13] and transgenic technologies may hinder field-testing because of regulatory and containment considerations. Given these limitations, the ability to use traditional mutagenesis techniques coupled to efficient targeting of genes would be advantageous. Fast neutron mutagenesis can generate small to medium size deletions in genomes. Li and colleagues exploited this strategy in rice to develop a PCR-based method that identified smaller-than-expected amplicons due to the presence of a deletion [14]. While this approach is potentially quite powerful, the mutants were rare and it is unclear if large populations with a sufficient density of medium sized deletions can be generated to make the process efficient. Further, deletions will most likely lead to a complete loss of gene function, making them unsuitable for studying the functions of essential genes.

Chemical mutagens have been used for forward genetic screens in a variety of organisms (see for example [15]). Compounds such as EMS and MNU induce single nucleotide changes by alkylation of specific nucleotides [16,17], resulting in mutations that are high in density and essentially randomly distributed [18]. Therefore, a relatively small population of individuals can provide an allelic series that includes a variety of missense changes with differing effects on protein function, and nonsense or splice site changes that cause truncation of the gene product. TILLING (Targeting Induced Local Lesions IN Genomes) [19] is a general reverse genetic technique that uses traditional chemical mutagenesis methods to create libraries of mutagenized individuals that are later subjected to high throughput screens for the discovery of mutations [20,21]. Mutations are identified by enzymatic cleavage of PCR amplified heteroduplexed DNA followed by band visualization using fluorescent end-labeling and denaturing polyacrylamide gel electrophoresis. The generality of the mutagenesis and the mutation discovery methods allow application of this approach to most organisms. Indeed, TILLING results have been reported for a variety of plants and animals [19,22-29]. The application of TILLING to rice, however, has been hampered by the difficulty in obtaining a population with a sufficiently high mutation density [27]. Here we describe progress in the TILLING of rice. Two different mutagenic treatments were used to generate two pilot scale mutant libraries, which were screened for mutations in 10 gene targets. A total of 57 nucleotide changes were identified. Based on this work, we conclude that a sufficient density of mutations can be obtained and detected by this method to create an efficient, large-scale mutation discovery service for rice.

## Results

### TILLING population development

**Figure 1:**
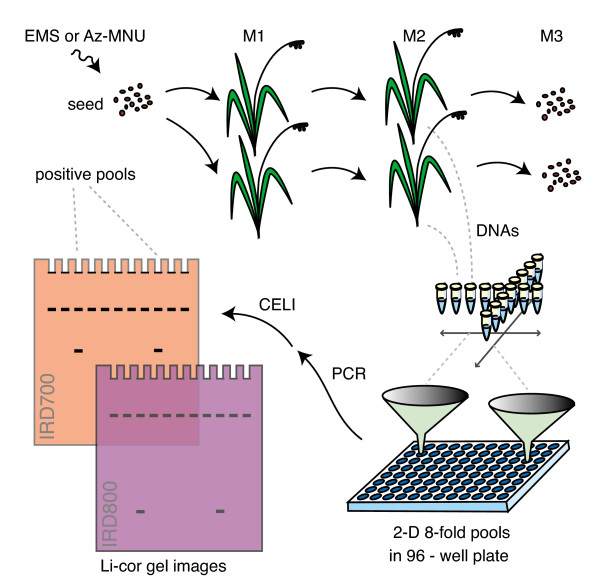
**TILLING strategy for rice**. After seed mutagenesis, chimeric M1 plants are allowed to self-pollinate and a single M2 plant is grown to provide DNA for mutation discovery and seed for banking. DNAs are pooled eightfold and arrayed in a two-dimensional format on 96-well plates. After PCR amplification of target genes, heteroduplexes are formed upon heating and annealing, and then digested using crude celery juice extract containing the CEL I nuclease. Cut strands are separated by denaturing polyacrylamide gel electrophoresis, and visualized by fluorescence detection using a Li-Cor DNA analyzer. The presence of cut products in two pools identifies the individual harboring the polymorphism.

To TILL rice, we chose a seed mutagenesis strategy analogous to what was used for TILLING Arabidopsis [19]. In this strategy, seeds are soaked with mutagen, and each fertile seedling defines a line harboring unique mutations (Figure 1). To avoid mosaicism in the M1 generation, plants are self fertilized and M2s are grown. Only one M2 individual from each M1 is selected for DNA extraction. This avoids sampling the same mutation in TILLING screens. Mutagenesis was performed using protocols that were previously established for grasses [30]. While EMS has been the mutagen of choice for many TILLING projects [19,22-28], a relatively low number of EMS-induced mutations were reported for the indica rice variety IR64 when treated with 0.8% and 1% EMS [27] and initial EMS treatments of the reference japonica variety Nipponbare resulted in insufficient seed germination due to toxicity (Tai and Colowit, unpublished observations). We therefore decided to test if alternative mutagens might provide a higher density of mutations with less toxicity to the treated seeds. A mutagenesis protocol using a combination of sodium azide plus methyl nitrosourea (Az-MNU) was selected. This mutagen has been previously used for forward genetic screens of barley [30]. A previously developed population in the japonica variety M-202 using 1 mM Az-7.5 mM MNU (Tai and Colowit, unpublished) was examined and shown to have a low mutation density (~1 per 1000 kb; Till and Cooper, unpublished results). Preliminary tests indicated that concentrations of MNU above 15 mM caused a large drop in germination and in viability of germinated plants (see Methods). Based on this, a combination of 1 mM Az-15 mM MNU was chosen for mutagenesis of Nipponbare. In addition, extended washing of seeds treated with EMS enabled the development of a Nipponbare population mutagenized with 1.5% EMS.

### Detection of mutations

**Figure 2:**
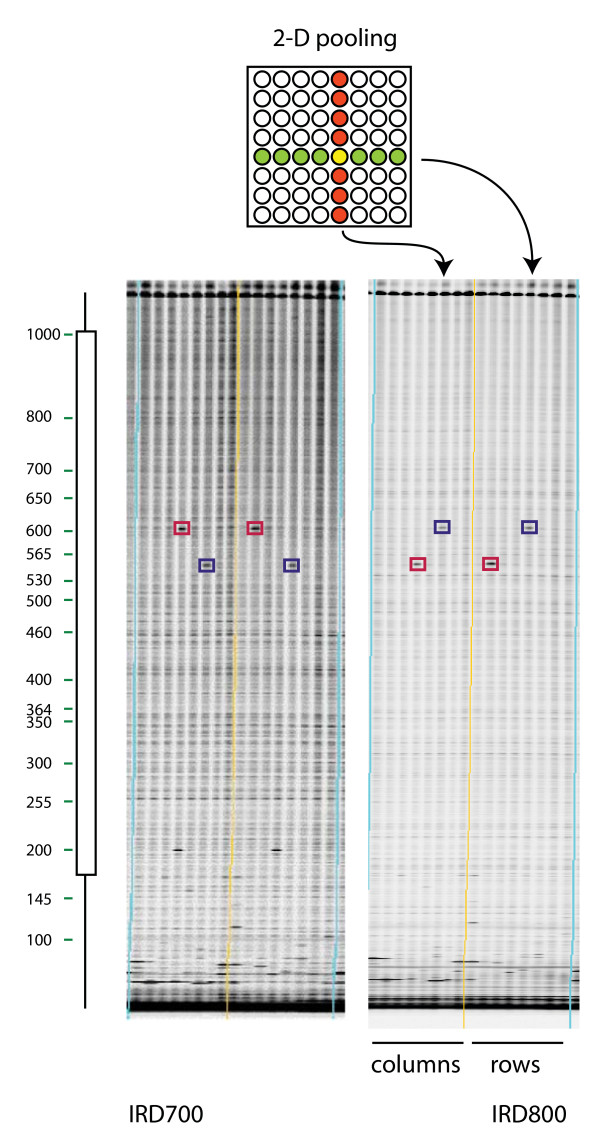
**Mutation discovery in eightfold pools of mutagenized individuals arrayed in a 2-D scheme**. Mutation discovery in eightfold pools of mutagenized individuals arrayed in a two-dimensional format. Top: A two dimensional pooling scheme is used whereby sixty-four samples from individual rice plants are first arrayed in an eight by eight grid. Samples from a common column are pooled in the first dimension and from a common row in the second dimension. Row pools and column pools are arrayed on adjacent lanes of an assay plate. Bottom: IRDye 700 (left) and IRDye 800 (right) gel images from the same sixteen lanes of a ninety-six lane TILLING assay for mutations in the 1,167 base pair OsDREB rice target. True mutations (boxed) produce a band in the column and row pool of each IRDye image, allowing the unique individual harboring the mutation to be determined in a single gel run (marked in yellow on the top diagram). Bands resulting from the same mutation are boxed a similar color. The molecular weight of the mutant band in the IRDye 700 image plus the molecular weight of the band in the IRDye 800 image adds up to the molecular weight of the full length PCR product. To the left of the images a virtual molecular weight ladder generated by the GelBuddy gel analysis program is shown. Next to this is the gene model (lines represent introns, boxes represent exons) that was generated when target primers were chosen using the CODDLe program.

For TILLING, we designed gene-specific primers that target selected regions of the rice genome (Table 1). Forward-strand primers were end-labeled with IRDye 700 dye and reverse-strand primers with IRDye 800 dye. We followed the standard high throughput TILLING protocol for mutation discovery [31]. After PCR amplification, products were denatured and annealed to form heteroduplexes between complementary strands. Heteroduplexes were then cleaved using a crude celery juice extract [32], and the products were size-fractionated on denaturing polyacrylamide gels using a Li-Cor DNA analyzer. Cleaved heteroduplexes produced two smaller molecular weight products, one labeled with IRDye 700 and the other with IRDye 800, whose sizes added up to the size of the full length product (Figure 2).

For both the Az-MNU and the EMS populations, 768 unique individual samples were selected for screening. We have previously reported robust discovery of mutations using pooled DNAs from eight individuals [18]. We therefore chose to pool samples eightfold for rice TILLING. Prior to pooling, genomic DNAs were normalized to a standard concentration. To shorten the time for validation of putative mutations, we used a two-dimensional arraying strategy whereby every individual is represented in two distinct eightfold pools and 384 unique individuals are screened in 96 lanes (2 × 8 × 96 = 384). A mutation will then produce signals in two gel lanes, whose positions allow deconvolution of the pools and provide the identity of the individual harboring the mutation (Figure 2). After discovery on a TILLING gel, the identity of each change was determined by sequencing.

**Figure 3:**
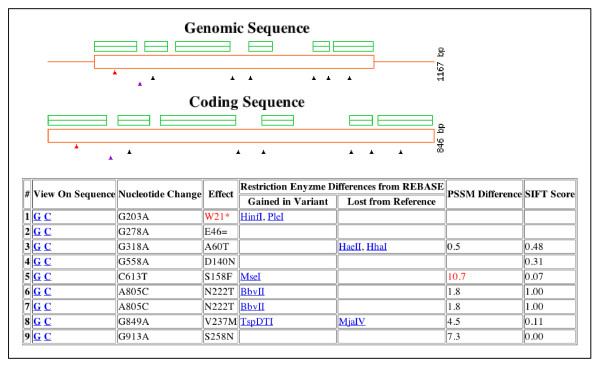
**PARSESNP output for the rice target OsDREB showing mutations discovered in both test populations**. At top, the gene model is shown in red with boxes corresponding to exons and lines for introns. Green boxes represent protein homology block alignments automatically generated using the SIFT program. Triangles indicate the location and type of mutation found. The red triangle denotes a nonsense mutation, the purple a silent change, and black triangles represent missense changes. The table summarizes the position and nucleotide change of each mutation, and the respective effect on the protein sequence (* indicates premature stop codon, = indicates a silent change, and letters indicate the amino acid according to single letter code). Restriction endonuclease sites that are either gained or lost due to the mutation are also listed and can be used for downstream genotyping. Missense changes are provided with PSSM and SIFT scores [46,47]. Scores are listed in red when the change is predicted to be damaging.

A total of 57 nucleotide changes were identified in the Az-MNU and EMS populations (Table 2). We obtained multiple mutations from each target. The mutations in OsDREB provide an example of an interesting allelic series that is anchored by a predicted severe loss of function (truncation due to a nonsense mutation at codon 21) and contains six other missense changes (Figure 3). To estimate the density of mutations for each population, we divided the total number of mutations identified by the total base pairs screened. We previously reported a diminished ability to detect mutations in the terminal 100 base pairs of each end of the amplicon [18]. Therefore, we subtracted 200 base pairs from the size of each amplicon to obtain the effective screening window size. To determine the total number of base pairs screened, we added the adjusted size of each amplicon and multiplied by the total number of samples screened. For each population, 7,951,104 base pairs were interrogated for mutations (10,353 bp × 768 individuals). We estimate a density of ~1 mutation/294 kb for the 1.5% EMS population and 1/265 kb for the population treated with Az-MNU. These mutation densities are ~threefold higher than have been previously observed for treatments with a lower concentration of mutagen [27] (B.T. and J.C., unpublished results).

We compared the mutational spectrum obtained through TILLING with the spectrum of natural polymorphisms identified by in silico comparison of sequences from japonica and indica varieties [33] (Table 3). In the case of chemically-induced mutations, frequent transition changes are expected because both EMS and MNU alkylate predominantly G residues and result in GC->AT changes [34,35]. Comparison of observed to expected ratios of GC->AT changes to all other changes indicated that these changes did not result from sampling natural variation (p < 0.01) as would be expected if the changes we observed had resulted from contamination. We conclude that most or all changes were induced through mutagenesis.

## Discussion

There are two critical steps in TILLING: mutagenesis and high throughput mutation discovery. For a successful reverse genetics service, it is paramount to achieve a satisfactory mutation density. The experience of the Seattle TILLING Project is that a library of mutagenized individuals is optimally suited for TILLING when in average at least one mutation is found per Li-Cor gel run. With samples pooled eightfold in a two dimensional array and an amplicon size of 1.5 kb, efficient TILLING requires a population with a mutation density of ≥ 1 mutation/500 kb. Mutation rates should increase with increasing mutagen concentration, but developing a well-mutagenized population can be challenging because diverse species and even varieties of the same species display a different response to mutagenic treatments [27]. For example, doses of mutagen that result in high lethality of the treated seed in one species, such as barley [36], may not yield the high mutation density that is seen with much less lethality in another species, such as Arabidopsis [37] or wheat [28]. We experienced such a difficulty with rice, where multiple attempts resulted in unsatisfactorily low mutation densities (Till, Tai, Leung, Henikoff and Comai, unpublished results). Although desirable, estimating the mutation rate from phenotypic analysis of M2 individuals is not simple. In Arabidopsis it is possible to reliably score embryo lethals, a phenotype resulting from deficiency at any of hundreds of genes. This measure has been incorporated in routine mutagenesis for TILLING [37]. Embryo lethality, however, is not as easily ascertained in rice and there is uncertainty on what other phenotypic indicators should be used. For example, the frequency of albino mutants is surprisingly high (8%) in rice populations that had a suboptimal mutation rate as determined by TILLING [27]. Through optimization of mutagenesis treatments and by choosing the highest mutagen concentration compatible with acceptable seed survival, we have succeeded in achieving a satisfactory mutation rate. Nipponbare populations treated either with 1.5 % EMS or by sequential soaking in 1 mM sodium azide and 15 mM MNU, showed a similar density of putative mutations detected by TILLING, ~1/300 kb. This mutation density is well suited to high-throughput mutation discovery. Comparison of phenotypic versus molecular mutation rates in these populations should provide phenotypic markers for assessing future TILLING populations.

The spectrum of observed changes differs from what was observed in Arabidopsis, maize, and wheat [24,28,37]. In these systems, EMS-induced changes were mostly GC->AT transitions, as expected from the frequent alkylation by EMS of guanine residues [16,38]. In our EMS-treated rice population, we found 70% GC->AT, 11% AT->GC, 4% GC->TA, and 15% AT->TA. This is not very different from the spectrum of mutations observed in a forward screen in Drosophila, which were respectively 76, 6 and 10 % (the balance were deletions) [39], and is consistent with the mutational spectrum reported by TILLING of barley [36]. Rice, therefore, might differ from Arabidopsis, maize, and wheat in its mutagenic response to EMS. There is less information on changes induced in plants by sodium azide and MNU, and no information on the combined action of the two mutagens. Both transitions (GC->AT, and AT->GC) and transversions were observed after sodium azide treatment in barley [40]. MNU induced predominantly GC->AT transitions in bacteria [34] and mice [35]. The analysis of the changes induced by both the EMS and Az-MNU treatment argued against the hypothesis that natural polymorphisms introduced by contamination of the Nipponbare population could be responsible for the observations. It is possible, nonetheless, that polymorphisms introduced through pollen or seed contamination might be responsible for a minority of the observed changes. This is the case in the Arabidopsis population used successfully by the Seattle TILLING Project where rare contaminants have been observed to introduce polymorphisms in multiple genes in the same individual [18,41]. In such cases, however, occasional concurrent mutations found either in the same gene of the same individual, or in other genes of the same individual are expected [18,29]. For example, based on the Poisson distribution, the probability of obtaining two mutations in the same individual of either EMS- or Az-MNU-treated populations after screening 10 genes × 1,300 bp of DNA is 0.95. An increasing number of concurrent mutations are of course less and less likely to be caused by the mutagenic treatment. Once an appropriate P-value threshold is crossed, usually requiring the screening of two or three dozen genes, these rare contaminants can be flagged and eliminated from subsequent TILLING searches. In the case of these populations, even if we assume conservatively that all concurrent or non-GC->AT changes are due to contamination, and remove them from the counts, the resulting mutation rates (EMS, 1/530 kb; Az-MNU, 1/497 kb) are still satisfactory and suitable for high throughput TILLING. A TILLING service for the rice community is planned and will be accessible at the following URL: .

## Conclusion

We have applied the TILLING method to the model crop rice and have identified two different mutagenic treatments that provide a suitably high density of mutations (≥ 1/500 kb) to consider developing rice for a high throughput TILLING service. From this pilot-scale experiment with 10 target genes, we were able to identify 57 nucleotide changes, most of which were inferred to be induced by mutagen treatment. One nonsense mutation and 29 missense mutations were identified, six of which are predicted to be damaging to protein function. The estimated mutation density is at least twofold greater than previously reported for rice and comparable to that reported for Arabidopsis.

## Methods

### Mutagenesis

The reference variety Nipponbare was selected for mutagenesis. Seeds from the original Nipponbare GA3 plant selected for use by the International Rice Genome Sequencing Project [3] were obtained from S. McCouch (Cornell University). These seeds were amplified in a greenhouse in Stuttgart, AR, followed by amplification in a field nursery in the same location and an additional amplification in a field nursery in Davis, CA. These seeds were subjected to the following mutagenesis protocols.

Sodium azide and methyl nitrosourea mutagenesis: For preliminary testing, we determined toxicity levels by treating with MNU as indicated below and germinating about 250 seeds. In two preliminary tests we observed respectively 49 and 56% germination (control displayed 72 and 80% germination) after treatment with 15 mM MNU. Higher concentrations of MNU resulted in progressively lower germination (20 mM, 36 and 48%; 30 mM, 24 and 23%). The fraction of viable (green) plants, however, was reduced precipitously by MNU concentrations above 15 mM (15 mM, 21 and 53%; 20 mM, 13 and 17%; 30 mM 13 and 14%). Therefore, 15 mM MNU was used as the highest concentration and administered in combination with sodium azide. A batch of ~10,000 seed (estimated by the average weight of a kernel) were placed in a 1L flask with 400 ml of ultrapure water and soaked overnight (~14–18 hours) at room temperature. Water was decanted and replaced with 325 ml of 1 mM sodium azide in sodium phosphate buffer (pH 3.5) and incubated at room temperature for 3 hours. Sodium azide was decanted and seeds were rinsed with 400 ml of ultrapure water (3 rinses, 5 minutes each). Following the third rinse, 400 ml of ultrapure water was added to the seeds for another overnight soak. Water was decanted and 400 ml of 15 mM MNU was added to the seeds followed by incubation for 3 hours at room temperature with occasional mixing. Rinsing with ultrapure water was performed as described, followed by rinsing under running tap water for 1 hour. Seeds were sown in flats and germinated in the greenhouse. Young seedlings (about 4 leaf stage) were transplanted by hand in a field nursery (Davis, CA). Plants were harvested and single panicles (M2 seeds) were randomly selected from each M1 plant. A single M2 plant from each M1 was grown for DNA analysis and M3 seed collection. 

EMS mutagenesis: A total of ~48,000 seeds (estimated by the average weight of a kernel) were treated in batches of 2,000. Batches were placed in a 250 ml flask and ultrapure water was added to about 1 cm above the seeds (~75 ml). Seeds were soaked overnight at room temperature for 24 hours. Water was decanted and 25 ml of 1.5% EMS (v/v) in water was added. Seeds were incubated for 18 hours at room temperature followed by decanting of the EMS and rinsing with 75 ml of ultrapure water (3 times, 5 minutes each) and 125 ml of ultrapure water (3 times, 20 minutes each). Seeds were then rinsed under running tap water for 2 hours before planting in flats. Seedlings (2 to 4 leaf stage) were transplanted to 98-cell plug trays and grown in the greenhouse until maturity. The M1 plants grown under these conditions typically produced one flowering tiller, which was harvested. A single M2 plant from each M1 was grown for DNA analysis and M3 seed collection.

### DNA extraction and sample pooling

Approximately 20 mg of lyophilized leaf tissue was used for DNA extraction with the FastPrep DNA kit (Qbiogene Inc/MP Biomedical, Irvine, CA, USA), as previously described [42]. Prior to pooling, the concentration of each sample was determined by agarose gel electrophoresis and comparison to lambda DNA references of known concentration (Invitrogen Inc, Carlsbad, CA, USA) with the aid of the UVP Bioimaging System, and LabWorks Image Acquisition and Analysis Software version 4.5 (UVP Inc, Upland, CA, USA). All samples were normalized to a standard concentration of 3 ng/μl and arrayed in an 8-by-8 grid. Two-dimensional pools were created by combining all samples in shared rows and all samples in shared columns as diagrammed in Figure 2.

### Primer design, high throughput TILLING, gel analysis, and mutation validation

Primers were selected using the CODDLe and Primer3 programs as previously described [37]. Forward and reverse primers were 5'-end labeled with IRDye 700 and IRDye 800 dye respectively. Custom synthesis of labeled and unlabeled primers was performed by MWG Biotech (Ebersberg, Germany). PCR, heteroduplex formation, heteroduplex digestion, and denaturing polyacrylamide gel electrophoresis were performed as previously described [42] with the following exceptions: 0.3 nanograms of pooled genomic DNA was used per PCR reaction, and 1 unit of crude celery juice extract was used per heteroduplex digestion reaction [32]. TILLING gels were analyzed with the aid of the GelBuddy semi-automated TILLING and Ecotilling gel analysis software [43]. Mutations identified by TILLING were validated by conventional sequencing as previously described [37]. The mutation rate was calculated by dividing the total number of observed mutations by the total surveyed DNA length (length of TILLED fragment × individuals surveyed). We subtracted 200 bp from each TILLED fragment to account for the difficulty in detecting CELI-cut DNA products that migrate in the top and bottom range of the gel. We have previously determined that less than 10% of the mutations expected to produce fragments that migrate in these ranges are identified [18]. Of the 57 scored mutations, 3 were in the range masked for the frequency calculation. Including the masked ranges in the calculation of total surveyed DNA decreases the mutation density from 1/294 to 1/351 kb for EMS and from 1/265 to 1/316 kb for Az-MNU.

### Statistical analysis

The probability of concurrent DNA changes in the same individual was calculated using the Poisson distribution [44]. The spectrum of natural polymorphism in rice was calculated using single nucleotide polymorphism (SNP) data obtained by the *in silico *comparison of japonica and indica rice sequences [33]. The first 62,051 SNPs involving base pair changes on chromosome 1 were counted using the Microsoft Excel COUNTIF function yielding 19,614 GC->AT, 21,778 AT->GC, 5,356 GC->TA, 4,233 GC->CG, 5,276 AT->TA, and 5,794 AT->CG. The corresponding fractions were 0.316, 0.351, 0.086, 0.068, 0.085, and 0.093. The expectations for natural polymorphisms were derived by multiplying the number of changes observed by TILLING times the measured natural fraction. The probability that the observed data fit the null hypothesis of natural polymorphisms was tested using a 2 × 2 table (e.g. for EMS: observed, 19 GC->AT and 8 others; expected 9 GC->AT and 18 others) and Fisher Exact test [45]. The P value for the same or stronger association was reported.

## Authors' contributions

TT planned and headed the development of the mutant populations. PC coordinated the experimental components of population development. BT and JC oversaw the high-throughput laboratory during DNA preparations, arrays and mutation detection. BT designed and tested the two-dimensional screening strategy. JC and EAG designed and tested the primers. BT, JC, EAG, SH, and LC interpreted the mutation detection data. SH and LC co-directed the high throughput STP laboratory. With TT, they conceived the project and obtained the funding for it. BT and LC were primarily responsible for drafting and revising the manuscript with contributions from co-authors. All authors read and approved the final manuscript.

## Figures and Tables

**Table 1:** Genes and primers

**Target name**	**Locus**	**Forward primer**	**Reverse primer**	**Amplicon size (bp)**
Os1433	Os02g36974	agccgtggtaatgaggatcgttgc	cctgaagccgcacacatggaattt	1,499
OsBZIP	Os01g64000	gtgagatggcatcggagatgagca	ctggctgccacccctatttgcatt	1,495
OsCALS8R	Os01g55040	cactcggcgtggaggaattacgac	aacactgcgaatctcccccagatg	999
OsDREB	Os01g07120	catcgtggcgcaacatgaaaaaga	ccacagtgcactcaacacacagtacaa	1,167
OsEXTE	Os10g33970	tgtttgccttccgttaatgccaca	agcgcccctaatccgaaccaaag	1,433
OsMAPK	Os07g38530	gccggaagcgttgtacaaggtcaa	cggcaagaaagcatttcaggcatc	1,495
OsPITA	Os12g18360	tggagttgttggccaaggaaatga	tttccagtccatttggggatgctg	1,008
OsR1A	Os05g41290	ggttctcatcggtcacgaccaaca	tccattgagaccgactgtgcaagg	751
OsRPLD1	Os01g07760	ggatggcttgatggcaacacatga	gtccttggggttcgcttcaattcc	997
OsTPS1	Os02g44230	agcggaaggtcccgcaataaggta	gcttcaaaattgtcgcctcggaaa	1,509

**TOTAL**				**12,353**

**Table 2:** Mutations discovered in the Nipponbare populations mutagenized with either 1.5% EMS or 1 mM Az-15 mM MNU

**Target**	**Nucleotide Change**	**Amplicon Position**	**Effect**^**a**^	**Population**		**Stock**
	
				**EMS**	**Az-MNU**	
Os1433	T->C	270	**L139P**^+^	X		TJ_0516
	T->A	304	D150E	X		TJ_0521
	G->A	326	**A158T**^+^	X		TJ_0160
	A->G	332	K160E	X		TJ_0160
	C->T	595	**S194F**^+^	X		TJ_0024
	G->T	950	intron	X		TJ_0042
	C->T	1144	intron		X	TT_0028
	G->A	1314	L213=	X		TJ_0478

OsBZIP	C->T	141	L46F		X	TT_0370
	G->A	475	S157N		X	TT_0846
	G->A	530	E175=	X		TJ_0747
	G->A	574	G190E		X	TT_0267
	G->A	640	G212D		X	TT_0150
	G->A	947	E314=		X	TT_0781
	G->A	1012	intron		X	TT_0150
	C->T	1083	L330=	X		TJ_0504

OsCALS8R	G->A	93	R291=	X		TJ_0461
	C->T	288	V356=	X		TJ_0521
	A->T	458	**Y413F**^+^		X	TT_0314
	A->G	574	I452V		X	TT_0044
	C->T	643	L475=		X	TT_0145
	G->A	843	E541=	X		TJ_0143

OsDREB	G->A	203	W21*		X	TT_0320
	G->A	278	E46=		X	TT_0781
	G->A	318	A60T		X	TT_0133
	G->A	558	D140N		X	TT_0627
	C->T	613	S158F		X	TT_0593
	A->C	805	N222T		X	TT_0150
	A->C	805	N222T		X	TT_0123
	G->A	849	V237M	X		TJ_0230
	G->A	913	S258N	X		TJ_0676

OsEXTE	C->T	73	intron		X	TT_0646
	A->G	282	E174G		X	TT_0682
	G->A	593	V278I	X		TJ_0122
	C->T	651	S297F	X		TJ_0024
	G->A	697	R312=		X	TT_0426
	A->G	1024	Q421=		X	TT_0419
	G->A	1093	P444=		X	TT_0503
	G->A	1164	C468Y	X		TJ_0155

OsMAPK	A->T	254	T331S		X	TT_0301
	T->C	799	G478=		X	TT_0399
	G->A	832	E489=		X	TT_0437
	A->T	1181	M606L	X		TJ_0360

OsPITA	G->A	293	D481N		X	TT_0515
	C->T	424	V524=	X		TJ_0183
	T->C	724	L624=	X		TJ_0517
	G->A	802	K650=	X		TJ_0162
	G->A	872	A674T	X		TJ_0025

OsR1A	C->T	217	T186I		X	TT_0246
	A->G	389	L243=		X	TT_0257

OsRPLD1	C->T	252	**T371I**^+^		X	TT_0643

OsTPS1	T->C	182	G182=		X	TT_0898
	C->T	364	intron	X		TJ_0710
	C->T	521	intron	X		TJ_0476
	A->T	598	**Q205H**^+^	X		TJ_0545
	G->A	1147	intron	X		TJ_0415
	A->T	1458	intron	X		TJ_0247

**Total**				**27**	**30**	

^a ^Synonymous (=), nonsense (*), and missense changes are shown, where the amino acid residue number is based on the exon-intron model for the TILLed fragment. Missense changes predicted by the program SIFT to be damaging to the encoded protein are indicated (+). Predicted damaging mutations are highlighted in bold.

**Table 3:** Analysis of natural and induced polymorphisms

**polymorphism**		**natural rate**^**b**^	**EMS**		**Az-MNU**	
		
**type**	**change**^**a**^		**ob**^**c**^	**ex**^**d**^	**ob**	**ex**
transitions	GC->AT	0.32	19	8.53	20	9.48

	**AT->GC**	0.35	3	9.48	6	10.53
transversions	**GC->TA**	0.09	1	2.33	0	2.59
	**GC->CG**	0.07	0	1.84	0	2.05
	**AT->TA**	0.09	4	2.30	2	2.55
	**AT->CG**	0.09	0	2.52	2	2.80
						

**Statistical analysis**^**e**^						

transitions	GC->AT	0.32	19	8.53	20	9.48

	**AT->GC**					
transversions	**GC->TA**					
	**GC->CG**	0.68	8	18.46	10	20.52
	**AT->TA**					
	**AT->CG**					

Probability			0.0067		0.0042	

^**a **^GC->AT is expected from G base-alkylating mutagens. Changes in bold are rare to uncommon in EMS- or MNU-treated organisms (see references in text). ^**b **^calculated from 62,000 natural SNPs of rice. ^**c **^observed. ^**d **^expected for natural polymorphisms. ^**e **^P of the null hypothesis that the observed changes are natural polymorphisms was calculated with Fisher Exact test for each for 2-by-2 table of data from the EMS (ethyl methanesulfonate treatment) and Az-MNU (sodium azide – methyl nitrosourea treatment). See Discussion and Methods for details.
